# Reproductive factors and breast cancer risk according to joint estrogen and progesterone receptor status: a meta-analysis of epidemiological studies

**DOI:** 10.1186/bcr1525

**Published:** 2006-07-19

**Authors:** Huiyan Ma, Leslie Bernstein, Malcolm C Pike, Giske Ursin

**Affiliations:** 1 Department of Preventive Medicine, Norris Comprehensive Cancer Center, Keck School of Medicine, University of Southern California, 1441 Eastlake Avenue #4407, Los Angeles, CA 90089, USA; 2 Department of Nutrition, University of Oslo, P.O. Box 1046, Blindern, 0316, Oslo, Norway

## Abstract

**Introduction:**

Although reproductive factors have been known for decades to be associated with breast cancer risk, it is unclear to what extent these associations differ by estrogen and progesterone receptor (ER/PR) status. This report presents the first meta-analysis of results from epidemiological studies that have investigated parity, age at first birth, breastfeeding, and age at menarche in relation to ER^+^PR^+ ^and ER^-^PR^- ^cancer risk.

**Materials and methods:**

We calculated summary relative risks (RRs) and corresponding 95% confidence intervals (CIs) using a fixed effects model.

**Results:**

Each birth reduced the risk of ER^+^PR^+ ^cancer by 11% (RR per birth = 0.89, 95% CI = 0.84–0.94), and women who were in the highest age at first birth category had, on average, 27% higher risk of ER^+^PR^+ ^cancer compared with women who were in the youngest age at first birth category (RR = 1.27, 95% CI = 1.07–1.50). Neither parity nor age at first birth was associated with the risk of ER^-^PR^- ^cancer (RR per birth = 0.99, 95% CI = 0.94–1.05; RR of oldest versus youngest age at first birth category = 1.01, 95% CI = 0.85–1.20). Breastfeeding and late age at menarche decreased the risk of both receptor subtypes of breast cancer. The protective effect of late age at menarche was statistically significantly greater for ER^+^PR^+ ^than ER^-^PR^- ^cancer (RR = 0.72 for ER^+^PR^+ ^cancer; RR = 0.84 for ER^-^PR^- ^cancer, *p *for homogeneity = 0.006).

**Conclusion:**

Our findings suggest that breastfeeding (and age at menarche) may act through different hormonal mechanisms than do parity and age at first birth.

## Introduction

Although it is well known that reproductive factors are associated with breast cancer risk [1-3], it is unclear to what extent these associations differ across subtypes of breast cancer defined by estrogen receptor (ER) and progesterone receptor (PR) status. There have been three narrative reviews of this topic [4-6]. The review published in 1986 [4] summarised the results from seven clinical case series and one hospital-based case-control study and did not find convincing evidence for any difference in effects of reproductive factors by ER status. The review published in 1993 [5] summarised the results from three population-based and four hospital-based case-control studies and concluded that nulliparity was positively associated with risk of ER^+ ^breast cancer but not with ER^- ^breast cancer. A review published in 2004 [6] summarised the epidemiological studies published by 2004 and concluded that nulliparity and delayed childbearing were associated with increased risk of ER^+ ^but not with ER^- ^cancer, whereas early age at menarche was more consistently associated with increased risk of ER^+^PR^+ ^cancer but not with ER^-^PR^- ^cancer. The 2004 review also stated that the protection from breastfeeding did not differ by ER/PR status, but no data were given [6].

The majority of the epidemiological studies reviewed had a small number of cases with receptor-negative cancer, and several important questions could not be addressed in these reviews. We recently conducted two large studies addressing these issues [7,8], and in the present study we report a meta-analysis conducted to quantitatively summarise studies that have investigated the association among parity, age at first birth, breastfeeding, or age at menarche in relation to ER^+^PR^+ ^and ER^-^PR^- ^breast cancer.

## Materials and methods

### Literature search strategy

We identified epidemiological studies (cohort or case-control studies) in MEDLINE from the year 1966 to Dec. 1, 2005, by running searches with the key words "Breast Neoplasm/ep [Epidemiology]" and "(ER or PR).mp [mp = title, abstract, name of substance, mesh subject heading]". We identified additional studies by tracking the references in all identified articles. We noticed that the studies published before 1995 all defined their receptor subtypes according to either ER or PR status and that most of them had a hospital-based study design, whereas most of studies published since 1995 used joint ER/PR status to define receptor subtypes and had a population-based study design. Using joint ER/PR status could reduce the chance of including any tumors in which one of the receptor statuses was mislabeled. Therefore, for inclusion into this meta-analysis, the identified articles have to have estimates of relative risk (RR) for ER^+^PR^+ ^and ER^-^PR^- ^breast cancer. We did not summarise the data for the two rare subtypes (ER^-^PR^+ ^and ER^+^PR^- ^breast cancer), because few studies reported estimates of RR for them. For exposure variables, we focused on the summary of results for reproductive factors that had been more frequently tested across studies, although some studies had also examined other factors such as body mass index, hormone replacement therapy, and so on. We thoroughly reviewed two cohort [9,10], five population-based case-control [7,11-14], and two hospital-based case-control studies [15,16] that investigated these issues. We also included one population-based case-control analysis in press [8] (Table 1).

### Meta-analysis

We extracted study-specific estimates of RR (odds ratios, rate ratios, and risk ratios) and their 95% confidence intervals (CIs) for highest versus lowest category of parity, age at first birth, breastfeeding, and age at menarche. For this analysis, we used RR to refer to any RR measure. Two studies presented their results by subgroups only, either by menopausal status [13] or by age at birth [10]. To obtain one summary estimate, we combined the RRs for the subgroups through weighting their log (base e) RRs by the inverse of their variances. For RRs of parity, seven studies [8-10,12-15] used nulliparous women as the reference group, whereas one of our own studies [7] used never-pregnant women as the reference group. For consistency with other studies, we re-computed our results to have nulliparous women as the reference group using our original data [7] and changed the lower limit in the highest category from five to three births. For the same reason, we also changed the lower limit in the highest category from 24 months of breastfeeding to 7 months for two of our own studies [7,8].

For parity, we also extracted or computed study-specific trend estimates. One study provided the estimates in the original publication [15]; we computed trend estimates using original data for two of our own studies [7,8] and computed trend estimates using RRs (and 95% CIs) for categorical variables in four studies [9,12-14] using the method described by Greenland and Longnecker [17]. We used the midpoint of each category in these calculations; for the open-ended highest category, we used its lower limit plus one as an estimate of the mean number of births. For the study of women under age 45 years from Georgia, Washington, or New Jersey, that reported RRs for ever versus never having given birth [12], we used two as our estimate of the number of births because that was close to the mean number of births for parous women under age 45 years in two of our own studies [7,8]. To calculate the estimate for one study, we combined the RRs given for one birth at age 20 and one birth at age 35, weighting their log RRs by the inverse of their variances [10].

To summarise the results by menopausal status, we did as follows. We accepted the definitions used in three studies [9,11,13]. Three studies restricted eligibility to young women (under age 40 [14], under age 45 [12], and under age 50 [8]). We combined these young women with premenopausal women to form a group representing women who were premenopausal or young.

We used fixed effects models to calculate summary RRs for all studies combined, by type of study design and menopausal status (premenopausal or young and postmenopausal status), because we did not detect statistically significant heterogeneity of effects between studies (*p *≥ 0.10) [18]. To ensure that each particular study contributed the same weight to the summary log RRs for ER^+^PR^+ ^and ER^-^PR^- ^subtypes, we used the inverse of the average variances of the two subtypes as the weighting variable. This is necessary for the calculated summary values to be directly comparable. In Tables 2, 3, 4, 5, we list the study-specific RRs (95% CIs), then the summary RRs (95% CIs) by type of study design and menopausal status, and the overall summary estimates for all studies.

To test for potential heterogeneity in risk by receptor status, we first calculated the average variance weighted difference in the log RRs for the two receptor status groups. The variance for each difference in log RRs is estimated by the sum of their variances. The differences are divided by their pooled variance, these weighted values are summed across studies, and the total is divided by the sum of the inverses of these pooled variances. To create a test statistic, χ^2 ^(with 1 degree of freedom), this value is squared and then divided by its variance, which is the inverse of the sum of the inverses of the pooled variances across studies. Potential heterogeneity in effect by study design and by menopausal status was examined by using standard homogeneity tests. All *p *values reported for homogeneity are two-sided, and *p *values less than 0.05 were considered statistically significant.

Using Egger's regression asymmetry test, we assessed the possibility of publication bias [19]. This analysis is based on a regression model in which the standard normal deviate is regressed against the study-specific estimate of the precision of log RR. When no publication bias is present, the points will scatter around a regression line that runs through the origin. We considered there to be publication bias if the intercept of the Egger's regression line deviated from zero with a two-sided *p *value of less than 0.10.

All analyses were performed using the Stata statistical software (Version 8; StataCorp LP, College Station, TX, USA).

## Results

### Characteristics of studies

Table 1 presents the basic characteristics of the ten studies that we reviewed. Nine of the ten studies were published between 1995 and 2005 [7,9-16], and the other is currently in press [8]. Seven of the ten studies are from the U.S. [7-12,16], and the others are from Canada [13], Australia [14], and Japan [15]. These studies include women of all ages. The main source of hormone receptor information was medical records [7-14]; in one study, a single laboratory provided the data on receptor status [16], and one study did not specify the source of their receptor data [15]. The percentage of participating cases with available ER/PR status was at least 65% in nine studies [7-14,16], whereas it was only 40% in one hospital-based case-control study [15]. The number of subjects included in these analyses ranged from 104 to 2,130 for ER^+^PR^+ ^and 80 to 1,081 for ER^-^PR^-^. On average, the number of ER^-^PR^- ^breast cancer cases involved in the analyses was approximately 46% of that of ER^+^PR^+ ^cancer cases. All studies considered confounding in their analyses although the confounders included in the models varied by study (Table 1).

### Parity

**Figure 1:**
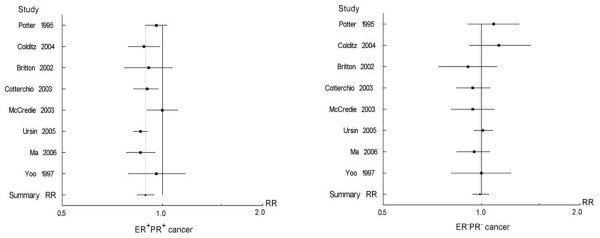
Parity and breast cancer risk by estrogen receptor/progesterone receptor (ER/PR) status: relative risk (RR) with corresponding 95% confidence interval per birth.

Eight studies were included in the meta-analysis of parity and breast cancer risk by ER/PR status (Table 2). Both the summary RRs for the highest versus the lowest category and the summary RRs per birth indicated that the protective effect of parity was confined to ER^+^PR^+ ^cancer. Each birth reduced the risk of ER^+^PR^+ ^cancer by 11% (RR per birth = 0.89, 95% CI = 0.84–0.94), and the *p *value for homogeneity between ER^+^PR^+ ^versus ER^-^PR^- ^cancer was less than 0.001 (Figure 1).

### Age at first birth

**Figure 2:**
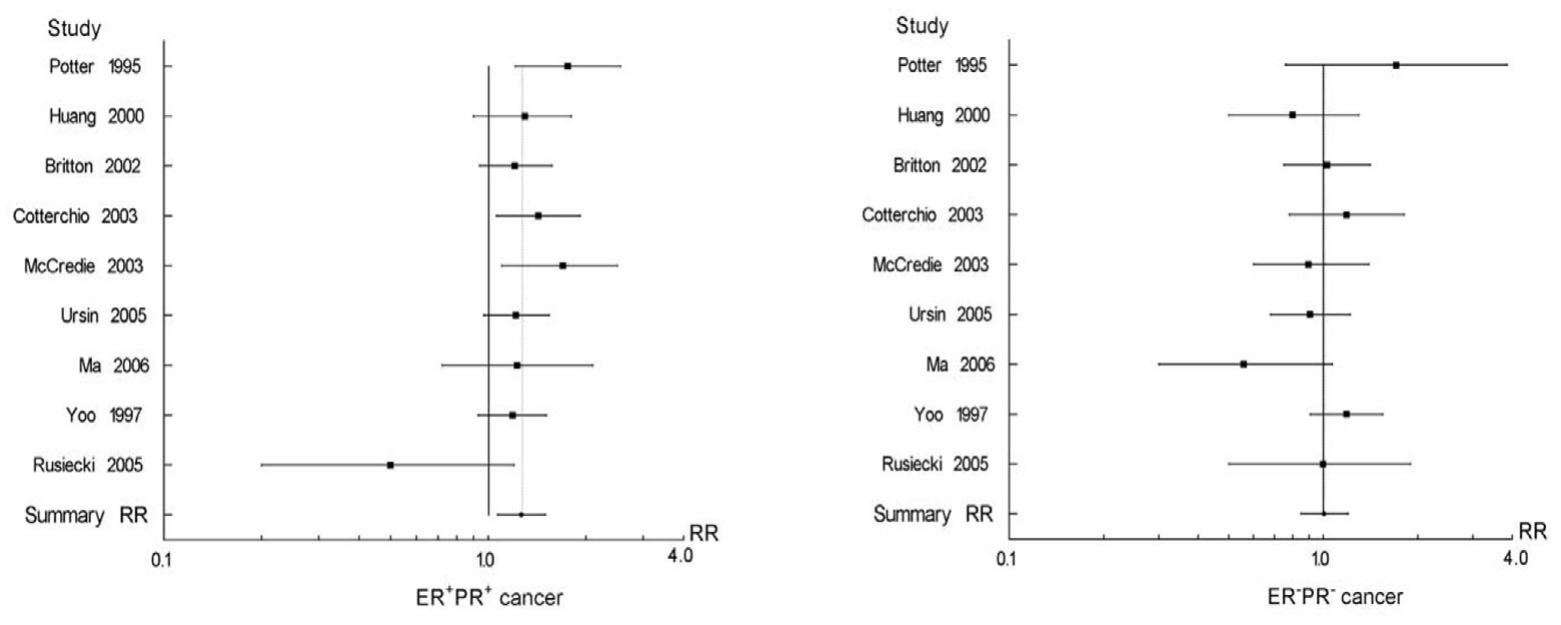
Age at first birth and breast cancer risk by estrogen receptor/progesterone receptor (ER/PR) status: relative risk (RR) with corresponding 95% confidence interval for oldest versus youngest age at first birth category.

Nine studies were included in the meta-analysis of age at first birth and breast cancer risk by ER/PR status (Table 3, Figure 2). Women in the oldest age at first birth category were on average at a 27% greater risk (summary RR = 1.27, 95% CI = 1.07–1.50) for ER^+^PR^+ ^cancer than women in the youngest age category, but age at first birth was not associated with risk of ER^-^PR^- ^cancer (summary RR = 1.01, 95% CI = 0.85–1.20). The difference in effects between ER^+^PR^+ ^and ER^-^PR^- ^was statistically significant (*p *for homogeneity between ER^+^PR^+ ^and ER^-^PR^- ^cancer = 0.010). The summary RR for ER^+^PR^+ ^cancer appeared greater among postmenopausal than premenopausal or young women (postmenopausal women: summary RR = 1.65, 95% CI = 1.15–2.38; premenopausal or young women: summary RR = 1.24, 95% CI = 0.96–1.62), but the difference was not statistically significant (*p *= 0.211).

### Breastfeeding

**Figure 3:**
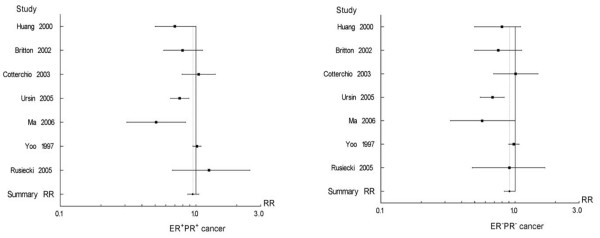
Breastfeeding and breast cancer risk by estrogen receptor/progesterone receptor (ER/PR) status: relative risk (RR) with corresponding 95% confidence interval for highest versus lowest breastfeeding category.

The summary RRs from the seven studies of breastfeeding show that breastfeeding was associated with reduced RRs of both ER^+^PR^+ ^and ER^-^PR^- ^cancer (summary RRs [95% CIs]: 0.95 [0.87–1.05] and 0.91 [0.83–1.00] for ER^+^PR^+ ^and ER^-^PR^- ^subtypes, respectively) (Table 4, Figure 3). The protective effect from breastfeeding was observed among population-based case-control studies, but not among hospital-based case-control studies. This difference is marginally significant (*p *= 0.071 for ER^+^PR^+ ^and *p *= 0.054 for ER^-^PR^- ^cancer). One reason for this discrepancy could be that the two hospital-based studies included a small number of cases and therefore had insufficient power to find any effect.

### Age at menarche

**Figure 4:**
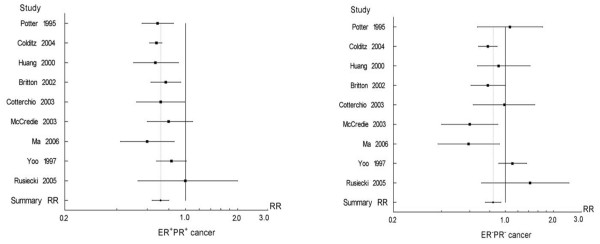
Age at menarche and breast cancer risk by estrogen receptor/progesterone receptor (ER/PR) status: relative risk (RR) with corresponding 95% confidence interval for oldest versus youngest age at menarche category.

Nine studies were included in the meta-analysis of age at menarche and breast cancer risk by ER/PR status (Table 5, Figure 4). The overall summary RRs of the oldest versus the youngest age at menarche category show that age at menarche was negatively associated with the risk of both ER^+^PR^+ ^and ER^-^PR^- ^cancer (summary RRs [95% CIs]: 0.72 [0.64–0.80] and 0.84 [0.75–0.94] for ER^+^PR^+ ^and ER^-^PR^- ^subtypes, respectively). The protective effect of late age at menarche was statistically significantly greater for ER^+^PR^+ ^than ER^-^PR^- ^cancer (*p *for homogeneity between ER^+^PR^+ ^and ER^-^PR^- ^cancer = 0.006).

### Publication bias

We found no evidence of publication bias in results across all studies for the factors we reviewed (Egger's test: all *p *> 0.10).

## Discussion

This quantitative overview estimates that each birth reduces the risk of ER^+^PR^+ ^breast cancer by 11% and that women who were in the oldest age at first birth category were, on average, at 27% higher risk of ER^+^PR^+ ^cancer than those who were in the youngest age at first birth category after adjustment for parity. Furthermore, we found that neither parity nor age at first birth was associated with reduced risk of ER^-^PR^- ^cancer. Breastfeeding and late age at menarche decreased the risk of both subtypes of breast cancer. The protective effect of late age at menarche was statistically significantly greater for ER^+^PR^+ ^than ER^-^PR^- ^cancer.

The most recent qualitative review of risk factors by ER/PR status was published by Althuis *et al*. in 2004 [6]. They reported that parity and age at first birth were associated with ER^+ ^but not ER^- ^breast cancer and that breastfeeding protected against both receptor-positive and -negative breast cancer. These results are consistent with the findings from our meta-analysis. However, Althuis *et al*. also concluded that late age at menarche was more consistently associated with reduced risk of ER^+^PR^+ ^breast cancer, whereas we found that late age at menarche protected against both ER^+^PR^+ ^and ER^-^PR^- ^breast cancer. The difference between our conclusion and that of Althuis *et al*. may be due partly to the quantitative nature of a meta-analysis, in which we weight the results of the studies by the precision of their estimates, and partly due to our incorporation of data from three more studies [8,10,16]. Two of these studies found that late age at menarche protected against both ER^+^PR^+ ^and ER^-^PR^- ^subtypes [8,10], whereas the other found no association with either subtype.

All the studies we summarised have been published, except for one study that is currently in press [8]. If studies that detected a difference in association by ER/PR status were more likely to be published, our results for parity and age at first birth could be biased. However, we found no evidence for publication bias for either parity or age at first birth results. If survival among cases depends on the two reproductive factors and differs between receptor-positive and receptor-negative tumors, this could result in bias among case-control studies. However, cohort studies also observed that the protective effect of parity [9,10] and early first birth [9] was restricted to ER^+^PR^+ ^cancer. We therefore think it is unlikely that survival bias explains why parity and age at first birth are associated with ER^+^PR^+ ^tumors, but not ER^-^PR^- ^tumors.

We also considered whether the different association by receptor status could be caused by residual confounding by age given that the ratio of ER^+^PR^+ ^to ER^-^PR^- ^subtypes increases with age [20,21]. We therefore examined the effect of parity using stratified analyses by age (5 years) from our own data [7]. We found that the protective effect from parity was still confined to ER^+^PR^+ ^cancer (results not shown). We therefore think it is unlikely that the difference in association by receptor status is due to residual confounding by age.

The main source of hormone receptor information for studies that we reviewed was medical records. Although we assume that the majority of laboratories have used immunoassays since 1995, we could not exclude the possibility that the assays and cutoffs for determining ER and PR status differed across studies. However, we believe that any such inconsistencies would be unlikely to cause the observed associations and, if anything, that they would bias the RR estimates toward the null value.

Some data suggest that compared with Caucasian women, African-American women are more likely to develop ER^-^PR^- ^cancer [20]. We were unable to address whether race modifies these associations, because only one study provided results by race [7]. However, in this study, we found that the associations for parity or breastfeeding were similar in Caucasian and African-American women.

The differences between the comparison and reference categories for age at first birth varied substantially across the studies, ranging from 1 [9,11,12,14,16] to 11 years [7,8] with a 4-year average. One would expect that the effects would be greater for the studies with the greater difference or gap, but this was not the case. Because we did not know the underlying distribution of age at first birth in each specific category from each study, we were unable to pursue this further.

The protective effects of a greater number of births and an early age at first birth against ER^+^PR^+ ^but not ER^-^PR^- ^breast cancer suggest that their effects influence risk predominantly through hormonal mechanisms that involve estrogen and progesterone. The effects of these hormones on breast tissue depend upon the amount of both hormones and their specific receptors [22-25]. A greater number of births and an early first birth may protect against receptor-positive breast cancer through several mechanisms: (a) by reducing estrogen and progesterone in plasma [26-28], (b) by increasing levels of sex hormone-binding globulin [26], or (c) by causing further differentiation of the breast epithelium, which may reduce the susceptibility to estrogen and progesterone [29].

Contrary to expectations, breastfeeding and late age at menarche protected against both ER^+^PR^+ ^and ER^-^PR^- ^subtypes, although menarche had greater protective effects against ER^+^PR^+ ^than did ER^-^PR^- ^cancer. This seems to be inconsistent with the hypothesis that these factors act through estrogen and progesterone mediated by their respective receptors [22-25]. However, evidence shows that when ER^+ ^progenitor cells are exposed to estrogen, they produce paracrine signals that cause neighbouring populations of ER^- ^cells to proliferate [30]. Thus, our findings do not preclude a hormonal mechanism for breastfeeding and late age at menarche but suggest that the mechanism differs from that involved in parity and age at first birth.

## Conclusion

Our quantitative overview shows that parity and early age at first birth protect only against ER^+^PR^+ ^breast cancer whereas breastfeeding and late age at menarche protect against both ER^+^PR^+ ^and ER^-^PR^- ^breast cancer. Our findings suggest that breastfeeding (and age at menarche) may act through different hormonal mechanisms than do parity and age at first birth.

## Abbreviations

CI = confidence interval; ER = estrogen receptor; PR = progesterone receptor; RR = relative risk.

## Competing interests

The authors declare that they have no competing interests.

## Authors' contributions

HM conducted the literature search and assembled the data, conducted the data analysis, and drafted the manuscript. LB revised the manuscript. MCP supervised the data analysis and revised the manuscript. GU provided advice on the literature search, data collection, and analysis and revised the manuscript. All authors have read and approved the final draft.

## Figures and Tables

**Table 1:** Basic characteristics of 10 studies

Study	Source of study subjects		Age (years)	Source of receptor information (percentage of subjects with the information)	Number of subjects		Adjustment for potential confounders in data analyses
					
	Cases	Controls			ER^+^PR^+^/ER^-^PR^- ^cases	Controls	
Cohort studies							
[9]	Iowa Women's Health Study, USA (7 years' follow-up, 1986–1992, 241,627 person-years)		55–69 at baseline	Medical record (65%)	414/80	-	BMI, BMI at age 18 years, WHR, age at menarche, type of MP, age at MP, oophorectomy history, FHBC, parity, age at first live birth, contraceptive, non-contraceptive estrogen use, ALC
[10]	Nurses' Health Study Cohort, USA (20 years' follow-up, 1980–2000, 1,029,414 person-years)		30–55 at baseline in 1976	Medical record (74%)	1,281/417	-	Age, age at menarche, time since menopause, parity at age, age at birth, FHBC, HBBD, years on ET, years on EPT, BMI, height, ALC
Population-based case-control studies							
[11]	North Carolina, USA	DMV/HCFA	20–74	Majority from medical record (91%)	381/262	790	Age at menarche, nulliparity/age at first full-term pregnancy, breastfeeding, abortion or miscarriage, BMI, WHR, OC, HT, FHBC, medical radiation to the chest, SMK, ALC, education, age, race
[12]	Georgia, Washington, and New Jersey, USA	RDD	20–44	Medical record (78%)	616/360	1,397	Age, race, education, BMI, WHR, parity, age at first birth, breastfeeding, OC, SMK, ALC, recreational exercise at age 12–13 and 1 year prior to interview, age at menarche, FHBC, MP, geographic site
[13]	Ontario, Canada	Assessment roll of the Ministry of Finance	25–74	Hospital laboratories and medical record (87%)	1,901/737	3,691	Age, age at menarche, parity, age at first live birth, OC, BMI, ALC, SMK, breastfeeding, HBBD, FHBC, current strenuous activity for pre-MP women; age at MP, HT, and oophorectomy history for post-MP women
[14]	Victoria and New South Wales, Australia	Electoral roll	<40	Medical record (81%)	323/181	564	Age, study center, study period, education, country of birth, marital status, FHBC, BMI, age at menarche, number of live births, OC
[7]	Georgia, Washington, Michigan, Pennsylvania, and California, USA	RDD	35–64	Medical record (82%)	2,130/1081	4,668	Age, race, education, FHBC, age at menarche, study site, number of full-term pregnancies and age at first full-term pregnancy only for models of parous women
[8]	California, USA	Neighbours	20–49	Medical record (84%)	854/385	440	Age, race, education, FHBC, age at menarche, gravidity, number of full-term pregnancies, OC, BMI, ALC, MP and HT, age at first full-term pregnancy and breastfeeding only for parous women
Hospital-based case-control studies							
[15]	Aichi Cancer Hospital, Nagoya, Japan	Hospital	≥25	Not specified (40%)	176/141	21,714	Age, occupation, FHBC, age at menarche, menstrual regularity as a teenager, age at MP, age at first full-term pregnancy, number of full-term pregnancies, breastfeeding, ALC, SMK
[16]	Yale, New Haven Hospital, USA	Hospital	40–80	Single laboratory (76%)	104/107	401	Age, race, FHBC, age at menarche, nulliparity/age at first full-term pregnancy, breastfeeding, MP, BMI, ever estrogen use, ALC, SMK

ALC, alcohol drinking; BMI, body mass index; DMV/HCFA, Division of Motor Vehicles for women under 65 years/Health Care Financing Administration for women aged 65 years or older; EPT, estrogen and progestin therapy; ER^+^, estrogen receptor-positive; ER^-^, estrogen receptor-negative; ET, estrogen therapy; FHBC, family history of breast cancer; HBBD, history of breast benign diseases; HT, hormone therapy; MP, menopausal or menopause; OC, oral contraceptive use; PR^+^, progesterone receptor-positive; PR^-^, progesterone receptor-negative; RDD, random digit dialing; SMK, cigarette smoking; WHR, waist-hip ratio.

**Table 2:** Parity and breast cancer risk by ER/PR status

Study	Subgroups by MP status	Exposure categories (*n*)	Highest/lowest exposure category	RR (95% CI)	
				
				ER^+^PR^+^	ER^-^PR^-^
Cohort studies					
[9]	Post-MP	3	≥3/Nulliparous	0.75 (0.52–1.06)	2.24 (0.69–7.24)
			Per birth	0.96 (0.89–1.03)	1.09 (0.91–1.30)
[10]	All women	3	4/Nulliparous	0.74 (0.61–0.89)	1.17 (0.80–1.70)
			Per birth	0.88 (0.79–0.98)	1.13 (0.92–1.41)
Summary RRs for cohort studies			Highest/Nulliparous	0.74 (0.50–1.10)	1.25 (0.84–1.87)
			Per birth	0.93 (0.80–1.08)	1.11 (0.95–1.29)
Population-based case-control studies					
[12]	Young	2	Ever/Nulliparous	0.83 (0.60–1.15)	0.82 (0.55–1.23)
			Per birth	0.91 (0.77–1.07)	0.91 (0.74–1.11)
[13]	All women	4	≥3/Nulliparous	0.62 (0.43–0.90)	0.77 (0.46–1.27)
			Per birth	0.90 (0.82–0.97)	0.94 (0.84–1.06)
	Pre-MP	4	≥3/Nulliparous	0.44 (0.26–0.75)	0.90 (0.46–1.76)
			Per birth	0.83 (0.73–0.94)	0.97 (0.83–1.14)
	Post-MP	4	≥3/Nulliparous	0.71 (0.53–0.97)	0.72 (0.46–1.12)
			Per birth	0.92 (0.86–0.98)	0.93 (0.84–1.03)
[14]	Young	4	≥3/Nulliparous	1.0 (0.5–1.2)	0.8 (0.4–1.3)
			Per birth	1.00 (0.90–1.11)	0.94 (0.81–1.09)
[7]	All women	4	≥3/Nulliparous	0.63 (0.54–0.73)	1.07 (0.86–1.32)
			Per birth	0.86 (0.82–0.90)	1.01 (0.95–1.08)
[8]	Young	4	≥3/Nulliparous	0.61 (0.42–0.88)	0.93 (0.60–1.44)
			Per birth	0.86 (0.78–0.95)	0.95 (0.84, 1.06)
Summary RRs for population-based case-control studies			Highest/Nulliparous	0.67 (0.55–0.82)	0.96 (0.79–1.17)
			Per birth	0.88 (0.83–0.94)	0.98 (0.92–1.04)
Hospital-based case-control study					
[15]		Per birth	Per birth	0.96 (0.79–1.17)	1.00 (0.81–1.23)
Summary RRs by MP status	Pre-MP/Young		Highest/Nulliparous	0.72 (0.52–0.98)	0.86 (0.63–1.18)
			Per birth	0.90 (0.82–0.98)	0.95(0.86–1.04)
	Post-MP		Highest/Nulliparous	0.72 (0.44–1.17)	0.86 (0.53–1.42)
			Per birth	0.93 (0.84–1.03)	0.97 (0.88–1.08)
Summary RRs for all studies			Highest/Nulliparous	0.75 (0.65–0.88)	1.01 (0.87–1.17)
			Per birth	0.89 (0.84–0.94)	0.99 (0.94–1.05)
*P *for homogeneity between ER^+^PR^+ ^and ER^-^PR^- ^cancer			Highest/Nulliparous	*p *<*0.001*	
			Per birth	*p *<*0.001*	

Test for homogeneity across all studies: *P*_ER+PR+ _= 0.52 and *P*_ER-PR- _= 0.77 for highest/nulliparous, *P*_ER+PR+ _= 0.84 and *P*_ER-PR-_= 0.80 per birth. Egger's test for publication bias for all studies: *P*_ER+PR+ _= 0.98 and *P*_ER-PR- _= 0.87. CI, confidence interval; ER^+^, estrogen receptor-positive; ER^-^, estrogen receptor-negative; ER/PR, estrogen and progesterone receptor; MP, menopausal or menopause; PR^+^, progesterone receptor-positive; PR^-^, progesterone receptor-negative; RR, relative risk.

**Table 3:** Age at first birth and breast cancer risk by ER/PR status

Study	Subgroups by MP status	Exposure categories (*n*)	Oldest/youngest age category	RR (95% CI) for oldest vs. youngest age category	
				
				ER^+^PR^+^	ER^-^PR^-^
Cohort study					
[9]	Post-MP	2	≥30/<30	1.76 (1.21–2.56)	1.71 (0.76–3.85)
Population-based case-control studies					
[11]	All women	2	>25/≤ 25	1.3 (0.9–1.8)	0.8 (0.5–1.3)
	Pre-MP	2	>25/≤ 25	1.0 (0.6–1.7)	0.9 (0.5–1.5)
	Post-MP	2	>25/≤ 25	1.6 (1.0–2.7)	0.9 (0.4–1.7)
[12]	Young	2	>24.3/≤ 24.3	1.21 (0.94–1.57)	1.03 (0.75–1.41)
[13]	All women	3	≥28/<24	1.43 (1.06–1.92)	1.19 (0.78–1.81)
	Pre-MP	3	≥28/<24	1.08 (0.73–1.60)	1.00 (0.60–1.65)
	Post-MP	3	≥28/<24	1.64 (1.28–2.10)	1.30 (0.89–1.89)
[14]	Young	2	≥25/<25	1.7 (1.1–2.5)	0.9 (0.6–1.4)
[7]	All women	4	≥30/<20	1.22 (0.97–1.54)	0.91 (0.68–1.22)
[8]	Young	4	≥32/<22	1.23 (0.72–2.10)	0.56 (0.30–1.07)
Summary RRs for population-based case-control studies				1.31 (1.07–1.60)	0.94 (0.76–1.15)
Hospital-based case-control studies					
[15]	All women	Per 5 years↑	5 years' increase	1.19 (0.93–1.51)	1.19 (0.91–1.55)
[16]	All women	2	≥30/<30	0.5 (0.2–1.2)	1.0 (0.5–1.9)
Summary RRs for hospital-based case-control studies				1.10 (0.78–1.55)	1.17 (0.83–1.65)
Summary RRs by MP status	Pre-MP/Young			1.24 (0.96–1.62)	0.92 (0.71–1.20)
	Post-MP			1.65 (1.15–2.38)	1.28 (0.89–1.84)
Summary RRs for all studies				1.27 (1.07–1.50)	1.01 (0.85–1.20)
*P *for homogeneity between ER^+^PR^+ ^and ER^-^PR^- ^cancer				*p *= *0.010*	

Test for homogeneity across all studies: *P*_ER+PR+ _= 0.80 and *P*_ER-PR- _= 0.70. Egger's test for publication bias for all studies: *P*_ER+PR+ _= 0.83 and *P*_ER-PR- _= 0.67. CI, confidence interval; ER^+^, estrogen receptor-positive; ER^-^, estrogen receptor-negative; ER/PR, estrogen and progesterone receptor; MP, menopausal or menopause; Per 5 years↑, Per 5 years' increase in age at first birth; PR^+^, progesterone receptor-positive; PR^-^, progesterone receptor-negative; RR, relative risk.

**Table 4:** Breastfeeding and breast cancer risk by ER/PR status

Study	Subgroups by MP status	Exposure categories (*n*)	Highest/Lowest exposure category	RR (95% CI) for highest vs. lowest category	
				
				ER^+^PR^+^	ER^-^PR^-^
Population-based case-control studies					
[11]	All women	2	Ever/Never	0.7 (0.5–1.0)	0.8 (0.5–1.1)
	Pre-MP	2	Ever/Never	0.7 (0.4–1.1)	0.7 (0.4–1.1)
	Post-MP	2	Ever/Never	0.8 (0.5–1.2)	1.1 (0.6–1.8)
[12]	Young	3	>12 months/Never	0.80 (0.58–1.12)	0.75 (0.50–1.12)
[13]	All women	3	>6 months/Never or nulliparous	1.05 (0.79–1.39)	1.01 (0.69–1.48)
	Pre-MP	3	>6 months/Never or nulliparous	1.41 (0.96–2.08)	0.86 (0.53–1.39)
	Post-MP	3	>6 months/Never or nulliparous	0.92 (0.73–1.16)	1.09 (0.77–1.52)
[7]	All women	4	>6 months/Never	0.76 (0.65–0.89)	0.68 (0.55–0.83)
[8]	Young	4	>6 months/Never	0.51 (0.31–0.84)	0.57 (0.33–1.00)
Summary RRs for population-based case-control studies				0.78 (0.64–0.94)	0.74 (0.61–0.89)
*P *for homogeneity between ER^+^PR^+ ^and ER^-^PR^- ^cancer				*p *= *0.80*	
Hospital-based case-control studies					
[15]	All women	Per 3 months↑	3 months' increase	1.02 (0.95–1.09)	0.98 (0.90–1.07)
[16]	40–80	3	≥12 months/Never	1.25 (0.67–2.50)	0.91 (0.48–1.67)
Summary RRs for hospital-based case-control studies				1.02 (0.92–1.14)	0.98 (0.88–1.09)
Summary RRs by MP status	Pre-MP/Young			0.83 (0.61–1.14)	0.73 (0.53–1.00)
	Post-MP			0.89 (0.62–1.27)	1.09 (0.77–1.56)
Summary RRs for all studies				0.95 (0.87–1.05)	0.91 (0.83–1.00)
*P *for homogeneity between ER^+^PR^+ ^and ER^-^PR^- ^cancer				*p *= *0.38*	

Test for homogeneity across all studies: *P*_ER+PR+_= 0.15 and *P*_ER-PR- _= 0.17. Egger's test for publication bias for all studies: *P*_ER+PR+ _= 0.18 and *P*_ER-PR- _= 0.18. CI, confidence interval; ER^+^, estrogen receptor-positive; ER^-^, estrogen receptor-negative; ER/PR, estrogen and progesterone receptor; MP, menopausal or menopause; Per 3 months↑, Per 3 months' increase in the duration of breastfeeding; PR^+^, progesterone receptor-positive; PR^-^, progesterone receptor-negative; RR, relative risk.

**Table 5:** Age at menarche and breast cancer risk by ER/PR status

Study	Subgroups by MP status	Exposure categories (*n*)	Oldest/Youngest age category	RR (95% CI) for oldest vs. youngest age category	
				
				ER^+^PR^+^	ER^-^PR^-^
Cohort studies					
[9]	Post-MP	2	≥13/<13	0.69 (0.56–0.85)	1.07 (0.67–1.71)
[10]	All women	2	15/11	0.68 (0.62–0.73)	0.78 (0.68–0.89)
Summary RRs for cohort studies				0.68 (0.59–0.79)	0.80 (0.69–0.93)
Population-based case-control studies					
[11]	All women	2	≥12/<12	0.67 (0.50–0.91)	0.91 (0.67–1.43)
	Pre-MP	2	≥12/<12	0.67 (0.4–1.0)	1.11 (0.67–1.67)
	Post-MP	2	≥12/<12	0.63 (0.42–1.0)	0.77 (0.45–1.43)
[12]	Young	2	≥13/<13	0.77 (0.63–0.94)	0.78 (0.61–1.00)
[13]	All women	4	≥14/<12	0.72 (0.52–0.99)	0.99 (0.63–1.53)
	Pre-MP	4	≥14/<12	0.49 (0.31–0.76)	1.12 (0.62–2.03)
	Post-MP	4	≥14/<12	0.84 (0.64–1.10)	0.94 (0.64–1.37)
[14]	Young		≥13/<13	0.8 (0.6–1.1)	0.6 (0.4–0.9)
[8]	Young	4	≥14/<12	0.60 (0.42–0.86)	0.59 (0.38–0.92)
Summary RRs for population-based case-control studies				0.73 (0.59–0.89)	0.77 (0.63–0.94)
Hospital-based case-control studies					
[15]	All women	Per 2 years↑	2 years' increase	0.83 (0.68–1.01)	1.11 (0.91–1.36)
16	All women	3	≥14/<12	1.0 (0.53–-2.00)	1.43 (0.71–2.50)
Summary RRs for hospital-based case-control studies				0.84 (0.64–1.10)	1.14 (0.87–1.49)
Summary RRs by MP status	Pre-MP/Young			0.71 (0.57–0.88)	0.77 (0.62–0.95)
	Post-MP			0.74 (0.54–1.01)	0.95 (0.70–1.30)
Summary RRs for all studies				0.72 (0.64–0.80)	0.84 (0.75–0.94)
*P *for homogeneity between ER^+^PR^+ ^and ER^-^PR^- ^cancer				*p *= *0.006*	

Test for homogeneity across all studies: *P*_ER+PR+ _= 0.95 and *P*_ER-PR- _= 0.23. Egger's test for publication bias for all studies: *P*_ER+PR+ _= 0.42 and *P*_ER-PR- _= 0.55. CI, confidence interval; ER^+^, estrogen receptor-positive; ER^-^, estrogen receptor-negative; ER/PR, estrogen and progesterone receptor; MP, menopausal or menopause; Per 2 years↑, Per 2 years' increase in age at menarche; PR^+^, progesterone receptor-positive; PR^-^, progesterone receptor-negative; RR, relative risk.
